# Immunohistochemical investigations and introduction of new therapeutic strategies in scleromyxoedema: Case report

**DOI:** 10.1186/1471-5945-4-12

**Published:** 2004-09-22

**Authors:** Frank Breuckmann, Marcus Freitag, Sebastian Rotterdam, Markus Stuecker, Peter Altmeyer, Alexander Kreuter

**Affiliations:** 1 Department of Dermatology, Ruhr-University Bochum, Gudrunstrasse 56, D-44791 Bochum, Germany

## Abstract

**Background:**

Scleromyxoedema is a rare chronic skin disease of obscure origin, which may often be associated with severe internal co-morbidity. Even though different casuistic treatment modalities have been described, to date, curing still seems to be impossible.

**Case presentation:**

We report a 44-year-old Caucasian female presenting with remarkable circumscribed, erythematous to skin-coloured, indurated skin eruptions at the forehead, arms, shoulders, legs and the gluteal region. Routine histology and Alcian blue labelling confirmed a massive deposition of acid mucopolysaccharides. Immunohistochemical investigations revealed proliferating fibroblasts and a discrete lymphocytic infiltration as well as increased dermal expression of MIB-1^+ ^and anti-mastcell-tryptase^+ ^cells. Bone marrow biopsies confirmed a monoclonal gammopathy of undetermined significance without morphological characteristics of plasmocytoma; immunofixation unveiled the presence of IgG-kappa paraproteins.

**Conclusions:**

Taking all data into account, our patient exhibited a complex form of lichen mxyoedematosus, which could most likely be linked a variant of scleromyxoedema. Experimental treatment with methotrexate resulted in a stabilisation of clinical symptoms but no improvement after five months of therapy. A subsequent therapeutic attempt by the use of medium-dose ultraviolet A1 cold-light photomonotherapy led to a further stabilisation of clinical symptoms, but could not induce a sustained amelioration of skin condition.

## Background

Lichen myxoedematosus (LM) represents a rare chronic skin disorder of unknown aetiology, which may often be accompanied by severe internal co-morbidity such as haematological involvement including paraproteinaemia, neurologic syndromes, gastrointestinal complications or cardiac abnormalities [1-4]. Clinically and histologically, LM is characterised by papular eruptions caused by an extensive dermal deposition of glycosaminoglycans [5]. Scleromyxoedema (SCL) is a variant of LM exhibiting erythematous, sclerotic and stiffed lesions beside lichenoid papules with only little tendency of spontaneous remission [6,7]. Even though various experimental treatment modalities have been described, to date, curing of SCL is still not possible.

The oncoming case presentation focuses on a progressive variant of SCL as referred to clinical, immunohistochemical and laboratory investigations, followed by low-dose methotrexate (MTX) and subsequent medium-dose ultraviolet A1 (UVA1) cold-light treatment.

## Case presentation

**Figure 1:**
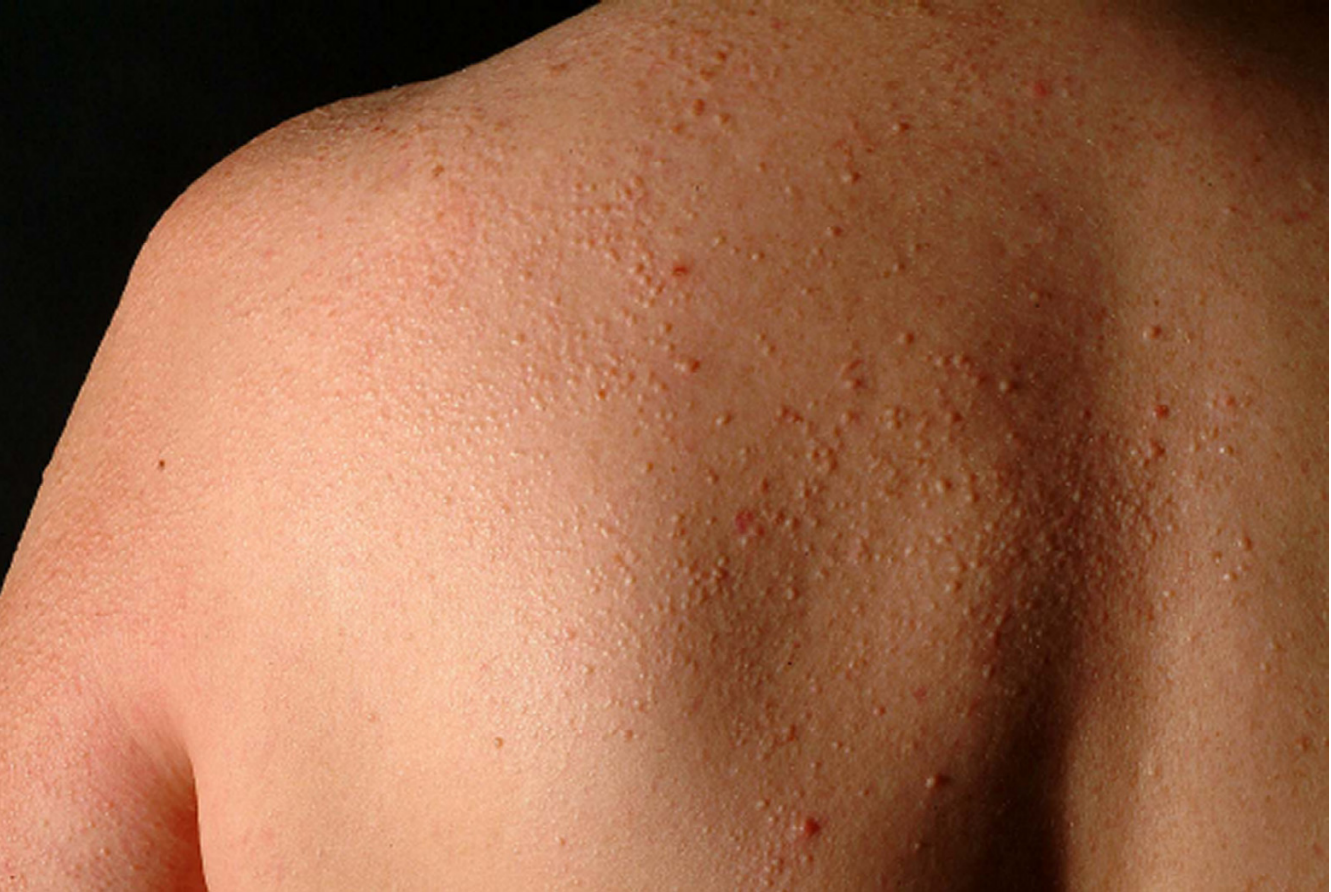
Lichenoid papules beside thickened skin on the dorsal aspect of the left shoulder.

We report a 44-year-old Caucasian woman who initially presented in 2003 with a multitude of progressive lichenoid 2–4 mm papules starting two years ago, particularly marked on the forehead right above both eyebrows, on the dorsal aspects of the forearms, shoulders, legs as well as on the gluteal region, accompanied by severe pruritus (Fig. 1). Clinically, the papules were judged as discrete, circumscribed, erythematous to skin-coloured, firm skin eruptions associated with an induration and stiffening of the affected lesions. Furthermore, the patient complained about a progressive thickening of the glabella. Even though we cannot ensure continuous clinical deterioration, at the time of the initiation of therapy, there was no hint for a beginning stabilisation or even improvement of symptoms. Otherwise, she felt healthy and well. The general examination was without pathological findings. Neither a topical nor a systemic therapy was yet applied.

Skin biopsies were taken from the left forearm/wrist, both legs and the left shoulder. Routine histological examination including haematoxylin-eosin, PAS labelling and Alcian blue staining revealed marked mucinous deposition within the upper and mid dermis beside an increased appearance of fibroblasts, collagen bundles and a discrete inflammatory infiltration.

**Figure 2:**
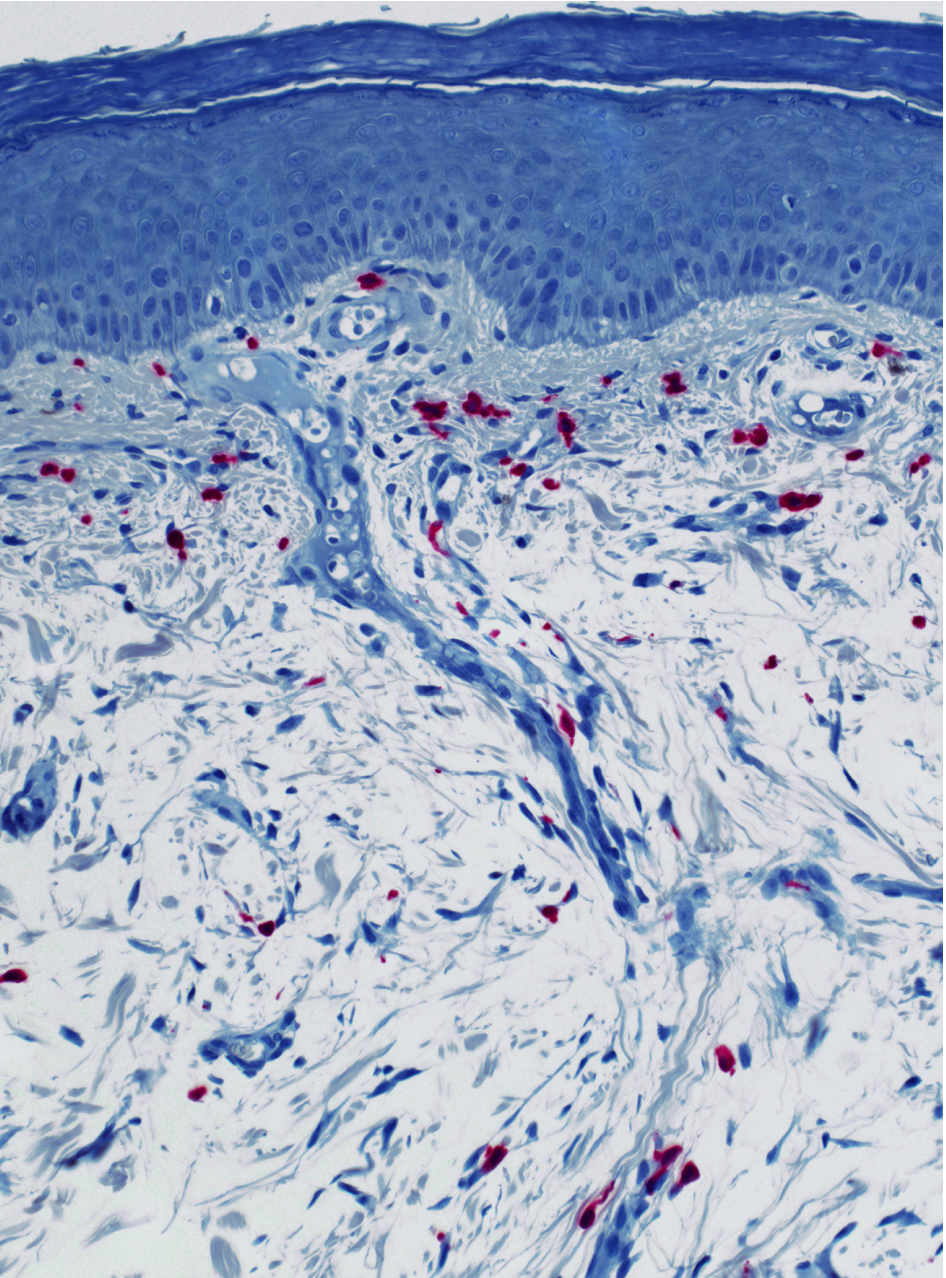
Immunhistochemistry unveiling sporadic lymphocytes beside a high number of anti-mastcell-tryptase^+ ^cells within the subepithelial perivascular infiltrate.

**Figure 3:**
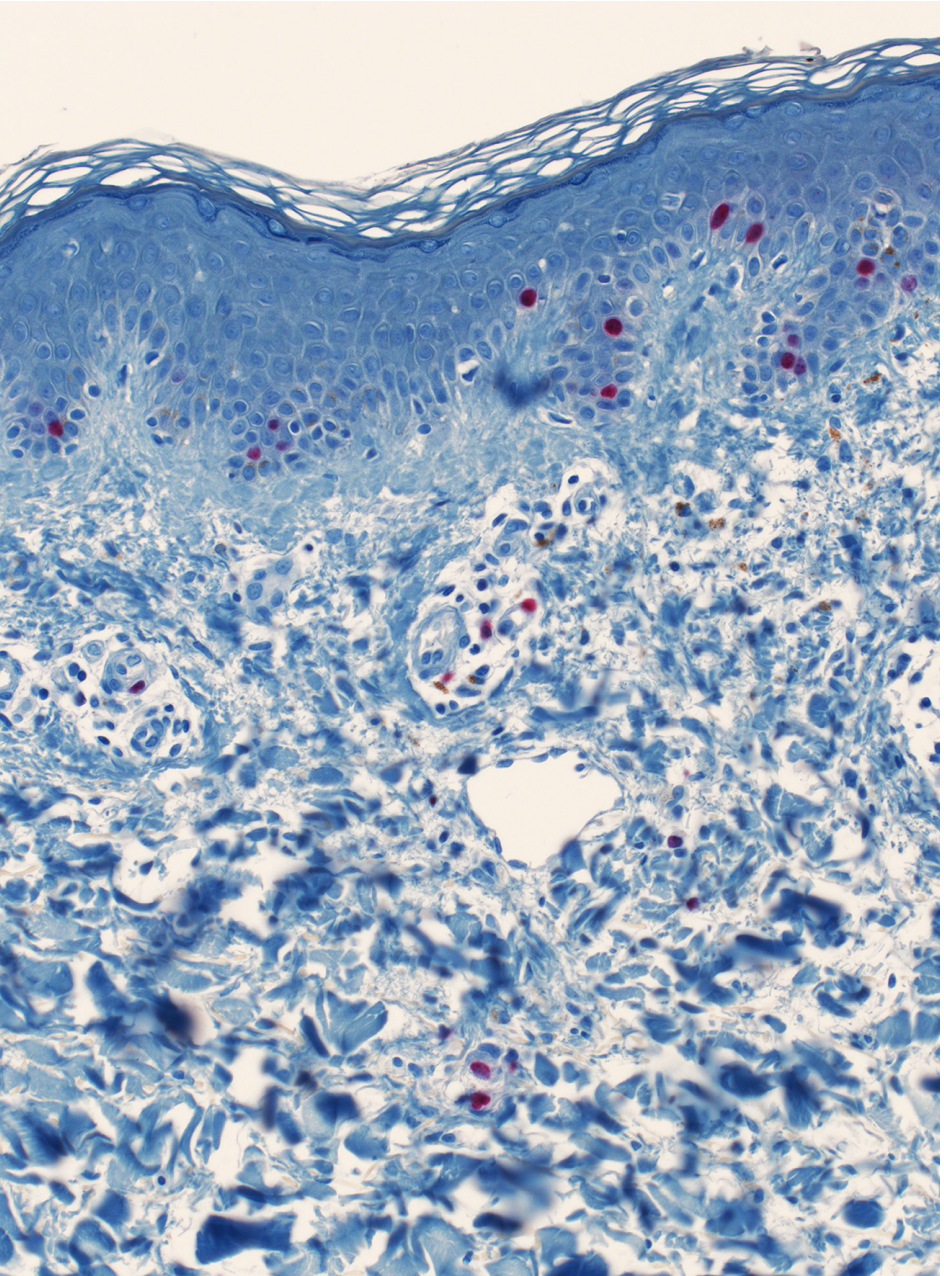
Mucinous deposition of the upper and mid-dermis accompanied by an elevated occurrence of MIB-1^+ ^dermal fibroblasts.

Additionally, immunohistochemical investigations were performed in order to enumerate CD4^+ ^(T helper cells), CD68^+ ^(macrophages), anti-mastcell-tryptase^+ ^(mastcells), decorin^+ ^(collagen fibril stability protein), MIB-1^+ ^(Ki-67^+ ^proliferating cells), CD20^+ ^(B lymphocytes), and FGF-R^+ ^(fibroblast growth factor receptor bearing cells) cells taking consecutive sections (Table 1). A punch skin biopsy measuring 3 mm in diameter was taken from affected skin of the right forearm. 5 μm paraffin-embedded sections were cut, mounted on slides and routinely preserved. Prior to the single immunolabelling, different pretreatments were performed for antigen retrieval (Table 1). The alkaline phosphatase anti-alkaline phosphatase (APAAP) technique using the labelled streptavidin-biotin (LSAB) method was used to enumerate immunopositive cells at an individual dilution (Table 1) taking consecutive sections. The alkaline phosphatase fast red detection kit utilised a biotinylated secondary antibody that binds to the primary antibody. This step was followed by the addition of an streptavidin enzyme conjugate binding to the biotin present on the secondary antibody. Afterwards the specific antibody-secondary-antibody-streptavidin-enzyme-complex was detected using a precipitating enzyme reaction product. Each step was incubated for a precise time and temperature. The alkaline phosphatase was used as enzyme; the chromogene fast red could be visualised. Cells were evaluated semiquantitatively (absent (-), rare (o), moderate (+), frequent (++)) directly below the dermoepidermal junction. Immunopositive cells were evaluated 'blind' separately in two view fields in a row (0.25 mm × 0.25 mm each) directly below the dermoepidermal junction resulting in a length of 0.25 mm and 0.50 mm in depth. In order to avoid a sampling error, a number of sections were randomly reevaluated by a second observer. In case of a significant difference, the sections had to be recounted by both observers. In brief, immunolabelling revealed occasional perivascular CD4^+ ^and CD20^+ ^lymphocytes located in the papillary dermis and a high number of anti-mastcell-tryptase^+ ^cells within the subepithelial perivascular infiltrate revealing a continuing decrease with increasing depth (Fig. 2). Simultaneously, an increased dermal expression of MIB-1^+ ^cells (Fig. 3), morphologically predominantly fibroblasts, within the upper and mid dermis and sporadic FGF-R^+ ^cells in an unspecific distribution could be detected. CD68 immunohistochemistry and intradermal decorin levels did not alter remarkably as compared to healthy controls (data not shown). Exact results of all immunohistochemical stainings are detailed in Table 2.

**Table 1:** Overview about the performed immunohistochemistry (alkaline phosphatase anti-alkaline phosphatase (APAAP) technique using the labelled streptavidin-biotin (LSAB) method)

Antibody	Source	Pretreatment*	Dilution	Incubation time
CD4	Novocastra Loxo, Dossenheim, Germany	H	1:60	30 min
CD68	Dako, Hamburg, Germany	P	1:25	30 min
Tryptase	Dako, Hamburg, Germany	P	1:400	28 min
MIB-1	Dako, Hamburg, Germany	H	1:10	32 min
FGF-R	Oncogene Research, San Diego, USA	N	1:10	30 min
Decorin	Oncogene Research, San Diego, USA	N	1:10	30 min
CD20	Novocastra Loxo, Dossenheim, Germany	H	1:50	30 min

*N = none, P = protease digestion, H = heat (microwave-3-step-technique)

**Table 2:** Semiquantitative data* of the immunohistochemical studies on a patient with scleromyxoedema

Antibody	CD4	CD68	Tryptase	MIB-1	FGF-R	Decorin	CD20
Upper dermis	+	o	++	+	o	-	o
Mid-dermis	-	-	+	+	-	-	-
Lower dermis	o	-	o	o	-	-	-

*- = absent, o = rare, + = moderate, ++ = frequent

Complete laboratory measurements unveiled the following pathological results: leucocytes 10870 μL^-1^, lymphocytes 13.2%, IgG 2000 mg/dl. There was no increase in B cell count. Immunoelectrophoresis disclosed albumin 51.4%, alpha-2 globuline 10.5%, gamma globuline 22.9%. Cranial x-ray, x-ray of the thorax, ultrasound of the abdominal organs, electrocardiography and urinary investigations were unremarkable. Blood smear cytological evaluation revealed beginning qualitative but still no quantitative changes as defined by leukocytic aberrations pointing towards a leftward shift. Serum immunofixation demonstrated an IgG-kappa paraproteinaemia. No elevation of the IgG-lambda paraprotein was assessed. Bone marrow biopsies displaying reactive lymphoid infiltration including minimal extension of plasma cells with monoclonal immunoglobuline production provided evidence for monoclonal gammopathy of undetermined significance (MGUS) without distinct morphological characteristics of a plasmocytic plasmocytoma or plasmoblasts.

Initially, 20 MHz ultrasound scanning producing cross section images of the skin was established in order to measure both structure and thickness of the skin at the dorsal aspects of the left wrist and the right forearm. The total thickness of the skin was measured from the entrance echo to the border between the dermis and the subcutaneous tissue. A cutaneous diameter of 2291 μm at the left wrist (lesional skin) and of 1106 μm at the right forearm (non-lesional skin) could be assessed.

In our unit, an experimental treatment modality using oral MTX 12.5 mg once per week followed by a subsequent folic acid application on the following day for a 6-months-period was subsequently initiated. MTX was well tolerated by our patient. After the first three months, the continuous progress of skin lesions during the last two years could be stopped and our patient experienced subjectively an improvement and objectively a stable clinical outcome without new lesions. Subjective impression of amelioration could not be confirmed by means of ultrasound measurement. Within the following two months, no further improvement could be evaluated, whereas no further progression such as formation of new lesions or increase of stiffness could be observed. Due to the unsatisfying clinical results and declining acceptance by our patient, MTX treatment was stopped and a subsequent therapeutic attempt with medium-dose UVA1 cold-light phototherapy was initiated. Irradiation consisting of 50 J/cm^2 ^single-dose UVA1 (CL 300000 liquid, Photomed, Hamburg, Germany) was performed four times a week for three weeks followed by two times a week for further two weeks resulting in a cumulative dose of 800 J/cm^2 ^after five weeks. Meanwhile, the skin status again remained stable, whereas no improvement could be observed. Therefore, our patient broke up phototherapy. To date, skin condition has slightly worsened without any current treatment modality.

## Conclusions

The population prevalence of SCL is known to be extremely low. Skin lesions are characterised by an increased deposition of acid mucopolysaccharides within the papillary and upper reticular dermis [8]. Even today, aetiology and pathogenesis remain hypothetic. Aberrant dermal deposition of monoclonal paraproteins predominantly of the IgG subtype combined with elevated IgG serum levels indirectly stimulating fibroblast activity are frequently found in LM patients [9-11]. Nevertheless, fibromucinous lesions of LM without the presence of paraprotein accumulations have also been described [12].

Beside typical skin eruptions, LM might also be associated with severe internal and neurological abnormalities such as cardiac irregularities, paralysis, hemiparesis or even progress to coma [3,4,13]. Despite sporadic case reports introducing new therapeutic strategies in LM and SCL, common treatment modalities are still disappointing and unsatisfactory. Topical treatment including hyaluronidase and triamcinolone as well as systemic efforts by the use of corticosteroids, cyclophosphamide, electron-beam therapy, hydroxychloroquine, PUVA, extracorporeal photopheresis, plasmapheresis or high-dose intravenous immunoglobulin partly displayed only limited success in individual patients [12,14-20].

In our patient, Alcian blue staining disclosed a remarkable deposition of mucinous material within the upper dermal layers combined with an increased appearance of proliferating MIB-1^+ ^and occasional FGF-R^+ ^fibroblasts in immunohistochemistry. Ki-67 (MIB-1) and fibroblast growth factors are involved in a variety of mitogen and proliferative activities [21]. Thus, the enhanced appearance of positive cells might represent an increased overall activation probably resulting in an aberrant release of mucopolysaccharides. Decorin contributes to the collagen fibril stability and high levels of decorin seem to be closely linked to dermal fibrotic stages as known from systemic sclerosis [22]. Here, an increased intradermal decorin expression could not be demonstrated. Simultaneously, in our patient the mucinous deposition was accompanied by a decreased presence of collagenous bundles. Interestingly, immunohistochemistry also revealed a number of CD4^+ ^and CD20^+ ^dermal inflammatory lymphocytes as well as anti-human mast cell tryptase^+ ^cells, which may profoundly contribute to mucinosis formation [23]. Unfortunately, we were not able to provide a longitudinal analysis of the different stainings due to missing consent of the patient to perform additional experimental biopsies within the course of therapy.

By considering clinical appearance, laboratory findings, immunofixation, bone marrow biopsy and histological evaluation, our patient presumably exhibited a complex variant of SCL. Fibroblast activity was supposed to be increased reflected by a corresponding high number of MIB-1^+ ^and FGF-R^+ ^cells within the upper dermis beside a massive mucinous deposition.

MTX therapy is approved in the treatment of malignant lymphoma. Additionally, low-dose MTX therapy has been established as a potent regimen in the treatment of T cell related skin diseases associated with a subsequent elevated fibroblast activation status (e.g. progressive systemic sclerosis) [24]. As in our patient paraproteinaemia of the IgG-kappa class, morphological signs of MGUS, proliferating dermal fibroblasts and a discrete T cell weighted lymphocytic dermal infiltration could be verified, we first decided to start a therapeutic attempt by the use of 12.5 mg MTX weekly in order to suppress local cellular activity following the promising reports about application of the anti-metabolite melphalan and the alkylating agent cyclophosphamide in previous studies [12,14]. Follow-up examinations were performed monthly. Three months after initiation of MTX therapy, an encouraging stable clinical outcome as well as an decline of pruritus without further progression of the disease was observed. However, the 5-months-follow-up revealed no apparent improvement of skin status leading to the joint decision of breaking up MTX treatment. UVA1 phototherapy has been shown to be effective in a number of inflammatory and fibrotic skin disorders by the induction of T cell apoptosis, collagenase activity and antiproliferative pathways [25]. Therefore, we decided to initiate a second attempt by the usage of a common medium-dose UVA1 irradiation protocol. Nevertheless, our patient broke up this regimen even after a 5-weeks-period due to the absence of immediate clinical improvement and an unfavourable time/benefit ratio, while follow-up examinations during this time revealed a stop of the progress anyway.

However, in order to interrupt the clinical progression or even therapy-resistance as reflected by our case presentation and by considering the stable skin conditions following UVA1 phototherapy, we are currently discussing a new therapeutic attempt applying extracorporeal photochemotherapy, as proposed by Krasagakis et al. [16], in order to stabilise or even improve the present slight aggravation without any potent therapy.

## List of abbreviations

LM: Lichen myxoedematosus; SCL: Scleromyxoedema; MTX: methotrexate; UVA1: ultraviolet A1; MGUS: monoclonal gammopathy of undetermined significance

## Competing interests

None declared.

## Authors' contributions

F.B. participated in the design of the study, carried out the immunohistochemistry, performed the statistical analysis and drafted the manuscript. S.R. carried out 20 MHz ultrasound scanning. A.K. conceived of the study. M.F., M.S. and P.A. participated in histology,  design and coordination.

All authors read and approved the final manuscript.

## Pre-publication history

The pre-publication history for this paper can be accessed here:


